# Risks of hospitalization and drug consumption in children and young adults with diagnosed celiac disease and the role of maternal education: a population-based matched birth cohort study

**DOI:** 10.1186/s12876-015-0415-y

**Published:** 2016-01-05

**Authors:** Cristina Canova, Gisella Pitter, Jonas F. Ludvigsson, Pierantonio Romor, Loris Zanier, Renzo Zanotti, Lorenzo Simonato

**Affiliations:** Laboratory of Public Health and Population Studies, Department of Molecular Medicine, University of Padua, via Loredan 18, 35131 Padua, Italy; Department of Medical Epidemiology and Biostatistics, Karolinska Institutet, 17177 Stockholm, Sweden; Department of Pediatrics, Örebro university Hospital, Örebro, 70185 Sweden; Friuli-Venezia Giulia Regional Health Information System, Informatica per il Sistema degli Enti Locali (INSIEL) S.p.A., Udine, Italy; Epidemiological Service, Health Directorate, Friuli Venezia-Giulia Region, Udine, Italy

**Keywords:** Celiac disease, Healthcare use, Maternal education, Birth cohort

## Abstract

**Background:**

Celiac disease (CD) may affect healthcare use in children and young adults. Socio-economic factors may act as a confounder or effect modifier. We assessed such hypotheses in a population-based birth cohort of young celiac subjects and references matched by maternal education.

**Methods:**

The cohort included all newborns recorded in the Medical Birth Register of Friuli-Venezia Giulia Region (Italy) between 1989 and 2011. CD incident cases were identified through pathology reports, hospital discharges and copayment exemptions and matched with up to five references by sex, year of birth and maternal education. Cox regression models were used to estimate Hazard Ratios (HRs) for major causes of inpatient diagnosis and drug prescription occurring after diagnosis in CD patients compared to references, stratifying by time of first event and maternal education.

**Results:**

We identified 1294 CD cases and 5681 references. CD cases had a higher risk of hospital admission for any cause (HR: 2.34; 95 % CI 2.08-2.63) and for all major ICD9-CM categories except obstetric complications, skin and musculoskeletal diseases, and injuries and poisoning. Prescription of all major ATC drug categories, except dermatologicals and genito-urinary medications, was significantly increased in CD subjects. For most outcomes, HRs were highest in the first year after CD diagnosis but remained significant after five or more years. HRs were similar across different categories of maternal education.

**Conclusions:**

Diagnosed CD subjects had a higher risk of hospitalization and medication use compared to the general population, even five or more years after diagnosis, with no effect modification of maternal education.

**Electronic supplementary material:**

The online version of this article (doi:10.1186/s12876-015-0415-y) contains supplementary material, which is available to authorized users.

## Background

Celiac disease (CD) is an immune-mediated small intestinal enteropathy triggered by gluten affecting approximately 0.5-1 % of the European population and frequently diagnosed during childhood [1].

Originally considered a rare malabsorption syndrome of childhood, CD is now recognized as a systemic disorder potentially affecting many organ systems beyond the gastrointestinal tract [2]. The clinical picture is variable and several complications and comorbidities can occur, including consequences of malnutrition (e.g., low bone mineral density, anemia), infections, neurological and autoimmune diseases (e.g. type I diabetes, thyroid disorders), [2, 3] and cancers such as lymphoma [4]. The only available treatment for CD is a life-long gluten-free diet. This dietary regimen is effective in controlling symptoms, however it is burdensome for the patient and his/her family and cannot prevent many complications and comorbidities of CD [2]. Moreover, children with CD, even if adherent to the gluten-free diet, often suffer from functional gastrointestinal disorders [5] and psychological disturbances [6] that may lead to impaired quality of life [7] and greater healthcare needs.

Few studies have investigated and reported an increased use of healthcare services and drugs and associated costs in adults with CD compared to the general population; [8–11] specific data regarding children and adolescents with CD are scarce. Furthermore, only one study [11] has so far assessed the long-term persistence of increased healthcare use after CD diagnosis.

Healthcare utilization pattern is affected by educational and socio-economic factors, which may reflect socio-economic differences regarding both morbidity and access to healthcare services. Currently, social determinants are emphasised as a major source of health inequalities within developed countries [12]. As far as we know, the impact of socio-economic status on healthcare utilization associated with CD has never been investigated.

Using a population-based birth cohort of subjects born between 1989 and 2011 and followed-up until the end of 2012, we examined the risk of first hospitalization and drug consumption at different time intervals from diagnosis in CD children, adolescents and young adults compared to reference individuals matched on age, gender and maternal education level. This study had three objectives: i) to assess whether children and adolescents with CD use more healthcare resources after diagnosis compared to subjects without CD of similar age, sex, and maternal education; ii) to assess whether the possible increased healthcare use is limited to the short period after CD diagnosis or persists long-term; iii) to assess whether the risk of healthcare use in CD subjects compared to unaffected subjects is modified by maternal education.

## Methods

### Setting and study population

We used a matched cohort design. The underlying study population consisted of 213 635 subjects (excluding abortions, stillbirths, and neonatal deaths within 30 days of birth) born in Friuli-Venezia Giulia (FVG) Region in Italy in the period 1989-2011 identified using the regional Medical Birth Register that contains socio-demographic data on the parents, details of the pregnancy, labour, and delivery, and the newborn at birth.

Italy has a tax-based National Health Service, which provides universal coverage to all Italian and European Union citizens resident in the country regardless of income, and it is organized at a regional level. FVG Region is covered by a regional integrated healthcare system developed in the 1980s with the goal of automatically collecting and pooling data on healthcare funded by the National Health Service using a unique regional identification code that has been described in detail previously [13].

The following Regional health data were used in this study (up to 2012): mortality records; pathology reports from all the pathology departments in the FVG Region, coded in the Systematized Nomenclature of Medicine (SnoMed); hospital discharge records collected during episodes of inpatient care occurring within the FVG Region or in Italy outside the Region with up to six diagnostic codes in the International Classification of Diseases ninth revision Clinical Modification (ICD9-CM); exemption from healthcare copayment coded in a national coding system; drug prescription records coded in the Anatomical Therapeutic Chemical (ATC) Classification System (available from 1995).

### Ethics, consent and permissions

The study was approved by the Institutional Review Board of the University of Padua (Italy). Patients’ data were completely anonymized and de-identified prior to analysis. Therefore, no informed consent and no Ethics Committee approval were required.

### CD definition

We defined CD as having at least one of the following [13]: a pathology report of villous atrophy (SnoMed codes D6218, D6318, M58, M58005, M58006, and M58007); a hospital discharge record with an ICD9-CM code 5790 in any diagnosis; an exemption from health-care copayment with code I0060 according to the Italian national coding system. In Italy, all patients with CD can obtain clinical tests and gluten-free food free of charge from the National Health Service, provided they have a CD diagnosis certified by a specialist on the basis of a biopsy report. CD onset was defined as the earliest date identifiable in the three sources of information (pathology, hospital and copayment exemption).

### Follow-up

We retrieved data on hospital discharges by cause of disease according to ICD9-CM classification (17 group of causes listed in Table 1) considering the main diagnosis and drug prescriptions according to first level of ATC Classification after date of diagnosis (index date in reference individuals).

**Table 1:** Risks of first hospital admission according to ICD9-CM classification after date of diagnosis in CD subjects compared to matched references^a^

ICD codes	Disease category	REFERENCES (*n* = 5681)		CD (*n* = 1294)		HR (95 % CI)
		*n*	py	*n*	py	
001–V89	Any	1245	33072	499	5915	**2.34** (2.08–2.63)
001–139	Infectious and parasitic diseases	73	40900	44	9396	**2.79** (1.89–4.12)
140–239	Neoplasms	56	41220	27	9686	**1.89** (1.16–3.08)
240–279	Endocrine. metabolic. immunity dis.	96	40936	69	9328	**3.19** (2.32–4.40)
280–289	Dis. of the blood and blood–forming organs	23	41334	25	9614	**4.47** (2.49–8.01)
290–319	Mental disorders	60	41203	35	9618	**2.52** (1.64–3.87)
320–389	Dis. of the nervous system and sense organs	114	40681	56	9414	**2.14** (1.53–2.98)
390–459	Dis. of the circulatory system	29	41405	16	9766	**2.77** (1.49–5.18)
460–519	Dis. of the respiratory system	347	38348	121	8763	**1.59** (1.28–1.98)
520–579	Dis. of the digestive system	220	40228	232	7846	**5.59** (4.57–6.83)
580–629	Dis. of the genito–urinary system	94	40861	42	9533	**1.95** (1.32–2.86)
630–679	Complications of pregnancy and puerperium	46	41394	15	9807	1.48 (0.82–2.67)
680–709	Dis. of skin and subcutaneous tissue	55	41215	16	9722	1.22 (0.68–2.17)
710–739	Dis. of the muscoloskeletal tissue	104	41056	27	9716	0.88 (0.55–1.44)
740–779	Congenital anomalies/Perinatal conditions	90	40902	44	9482	**2.18** (1.50–3.17)
780–799	Ill-defined conditions	83	40898	55	9413	**2.94** (2.06–4.19)
800–999	Injuries and poisoning	167	41491	57	9840	1.35 (0.98–1.86)
V01–V89	Factors influencing health status	58	41092	41	9468	**2.94** (1.94–4.47)

*Py* person-years, *CD* celiac disease, *HR* Hazard Ratio, *CI* confidence interval, *Dis*. Disease

Figures in bold are statistically significant results (*p*-value <0.05)

^a^Matched by year of birth, gender and maternal education

### Data analysis

For each case with a CD diagnosis up to five reference individuals were selected from subjects in the Medical Birth Register alive on the date of CD diagnosis (index date), matched on gender, year of birth and maternal education levels (up to middle school; secondary school; university level). Matching was implemented to control for confounding and improve statistical efficiency and to be able to attribute the beginning of follow-up (index date) that corresponds to CD diagnosis to reference individuals. Follow-up started at 30 days after the index date (to exclude hospitalizations and prescriptions concomitant to CD diagnosis) until the earliest of: end of the study (December 31, 2012), death, migration out of the area, or first hospitalization for each group of causes/first drug prescription for each ATC level. Concerning drug prescription outcomes, analyses were restricted to subjects with index date ≥1995 (because drug prescription data are available from that year). Cox regression models were used to estimate the Hazard Ratios (HRs) and 95 % confidence intervals (CI) for each of the healthcare use outcome in patients with CD compared to reference individuals within the same stratum. The assumption of proportional hazards was investigated by studying graphs over the log cumulative hazards function and the Shoenfeld residuals, and verified by a global test of rho. Analyses were also stratified by time of first hospital admission/drug prescription, looking at outcomes occurred between 30-365 days and >365 days after the index date. For the latter analyses only subjects with at least one year of follow-up (alive and resident in the area one year after the index date and without any hospitalization/drug prescription for each ICD9-CM/ATC category within one year from the index date) were considered. In order to assess the possible long-term effect on healthcare use in CD individuals compared to references, we examined the risk of hospitalization and drug consumption five or more years after diagnosis. For this analysis follow-up started five years after the index date and only subjects alive and resident in the area at this time were included. In this analysis, we included subjects with hospitalization/drug prescription within five years from the index date.

In order to evaluate a possible modifying effect of socioeconomic status, analyses were subsequently stratified by maternal education levels.

## Results

We identified 1294 subjects with CD and 5681 reference individuals. Biopsy confirmation was available for 920 (71 %) CD individuals; 179 (14 %) were instead identified by hospital discharges and copayment records, 112 (9 %) only by hospital discharge records and 83 (6 %) only by copayment records. Mean age at diagnosis (age at index date for references) was 7.2 ± 5.3 years (min 0, max 23.8 years). Analyses of drug prescriptions were restricted to 1215 CD subjects diagnosed after 1995 and 5366 matched references. 1166 CD cases (5100 references) were alive and resident in the Region one year after the index date and 777 (3316 references) 5 years after.

CD subjects were at increased risk of hospital admission for any cause (HR: 2.34; 95 % CI: 2.08-2.63) and for all major groups of causes according to ICD9-CM classification, except for complications of pregnancy and puerperium, diseases of skin and subcutaneous tissue, diseases of the musculoskeletal tissue, and injuries and poisoning (Table 1). The risk of drug prescription was also significantly increased in CD cases for all ATC categories except dermatologicals (ATC = D) and genito-urinary medications and sex hormones (ATC = G) (Table 2).

**Table 2:** Risks of first drug prescription according to first level of Anatomical Therapeutic Chemical (ATC) Classification after date of diagnosis in CD subjects compared to matched references^a^

ATC codes	Drug category	REFERENCES (*n* = 5366)		CD (*n* = 1215)		HR (95 % CI)
		*n*	py	*n*	py	
A	Alimentary tract and metabolism	537	38094	298	8054	**2.55** (2.19–2.97)
B	Blood and blood forming organs	258	40354	193	8846	**3.69** (3.02–4.50)
C	Cardiovascular system	102	40676	43	9511	**1.77** (1.22–2.57)
D	Dermatologicals	180	40060	55	9381	1.22 (0.88–1.69)
G	Genito–urinary system and sex hormones	126	40749	38	9629	1.22 (0.83–1.79)
H	Systemic hormonal preparations. excluding sex hormones and insulins	778	37614	316	8082	**2.06** (1.79–2.37)
J	Antiinfectives for systemic use	3660	17612	885	3680	**1.27** (1.17–1.39)
L	Antineoplastic and immunomodulating agents	19	41350	25	9697	**5.24** (2.82–9.73)
M	Musculo–skeletal system	170	40674	73	9436	**1.93** (1.44–2.59)
N	Nervous system	156	40703	56	9533	**1.53** (1.12–2.11)
P	Antiparasitic products. insecticides and repellents	216	40259	90	9271	**2.01** (1.56–2.60)
R	Respiratory system	2059	27958	560	6004	**1.33** (1.20–1.48)
S	Sensory organs	147	39939	51	9252	**1.42** (1.02–1.99)

Py: person-years; CD: celiac disease; HR: Hazard Ratio; CI: confidence interval

Figures in bold are statistically significant results (*p*-value <0.05)

^a^Matched by year of birth, gender and maternal education; analysis restricted to subjects with index date ≥1995 (because drug prescription data are available from that year)

The risk of any hospital admission in CD subjects compared to references was higher during the first year after diagnosis but remained statistically significant also after the first year of follow-up (Table 3). Examining single major groups of causes, the chronological pattern was heterogeneous (Table 3): for endocrine/metabolic/immune disorders and digestive disorders HRs were higher during the first year and declined afterwards; a similar pattern was seen, although less evident, for infectious, respiratory, and congenital disorders, for ill-defined conditions and factors influencing health status. On the contrary, hospitalization for nervous/sensory disorders and genito-urinary disorders had similar HRs within one year from diagnosis and after one year. Finally, concerning blood, mental and circulatory disorders, the low number of events occurring during the first year did not allow to estimate hazard ratios. Five years after diagnosis, CD subjects still had a higher risk of being admitted to the hospital for any cause (HR: 2.03; 95 % CI: 1.71-2.41) and for infectious (HR: 2.45; 95 % CI: 1.24-4.85), endocrine/metabolic/immune (HR: 3.13; 95 % CI: 1.94-5.06), mental (HR: 1.99; 95 % CI: 1.04-3.81), nervous/sensory (HR: 2.76; 95 % CI: 1.62-4.72), circulatory (HR: 2.39; 95 % CI: 1.14-5.00), digestive (HR: 2.90; 95 % CI: 2.09-4.02), and congenital disorders (HR: 2.93; 95 % CI: 1.64-5.21), as well as for ill-defined conditions (HR: 2.62; 95 % CI: 1.50-4.61) and factors influencing health status (HR: 4.87; 95 % CI: 2.32-10.23) (Additional file 1: Table S1).

**Table 3:** Risks of first hospital admission according to ICD9–CM classification after date of diagnosis in CD subjects compared to matched references, stratified by first outcome occurrence (30–365 days after the index date/>365 days after the index date)^a^

First outcome occurrence	30–365 days					>1 year after index date^b^				
ICD codes	REFERENCES		CD		HR (95 % CI)	REFERENCES		CD		HR (95 % CI)
	*n*	py	*n*	py		*n*	py	*n*	py	
001–V89	281	5260	243	1095	**4.28** (3.56–5.13)	964	27812	256	4820	**1.58** (1.36–1.85)
001–139	18	5380	17	1221	**4.38** (2.25–8.55)	55	35520	27	8176	**2.23** (1.37–3.63)
140–239	8	5384	6	1226	2.88 (0.94–8.85)	6	35836	21	8459	**1.72** (1.00–2.98)
240–279	12	5383	24	1216	**8.37** (4.12–16.99)	84	35553	45	8112	**2.42** (1.66–3.52)
280–289	7	5426	13	1309	--	16	35947	12	8392	**2.70** (1.21–6.04)
290–319	12	5476	9	1272	--	48	35819	26	8394	**2.26** (1.37–3.72)
320–389	32	5375	13	1224	1.83 (0.94–3.56)	82	35306	43	8190	**2.26** (1.54–3.32)
390–459	2	5388	0	1229	--	27	36017	16	8537	**2.99** (1.59–5.64)
460–519	66	5356	38	1213	**2.62** (1.74–3.95)	281	32991	83	7550	**1.34** (1.03–1.73)
520–579	30	5375	123	1156	**18.96** (12.52–28.70)	190	34853	109	6690	**3.07** (2.38–3.97)
580–629	23	5378	11	1226	1.92 (0.88–4.19)	71	35483	31	8307	**1.95** (1.25–3.05)
630–679	1	5388	0	1229	--	45	36006	15	8578	1.57 (0.87–2.82)
680–709	6	5385	2	1228	0.81 (0.10–6.70)	49	35830	14	8493	1.27 (0.69–2.32)
710–739	12	5384	7	1226	2.32 (0.87–6.21)	92	35672	20	8490	0.70 (0.39–1.23)
740–779	21	5381	16	1220	**3.39** (1.74–6.59)	69	35521	28	8262	**1.80** (1.14–2.85)
780–799	16	5381	18	1220	**5.43** (2.76–10.66)	67	35517	37	8193	**2.33** (1.52–3.56)
800–999	1	5389	1	1229	5.00 (0.31–79.95)	166	36102	56	8611	1.33 (0.97–1.83)
V01–V89	15	5379	15	1223	**4.18** (2.00–8.74)	43	35713	26	8245	**2.50** (1.50–4.17)

*Py* person–years, *CD* celiac disease, *HR* Hazard Ratio, *CI* confidence interval, Figures in bold are statistically significant results (*p*-value <0.05)

^a^Matched by year of birth, gender and maternal education; ^b^excluding subjects with less than one year of follow-up for each outcome

The risk of drug prescription was higher in the first year after diagnosis for all ATC groups, except for systemic anti-infectives (ATC = J) and respiratory system (ATC = R) medications, which showed a stable risk over time (Table 4). Risk estimates for cardiovascular drugs (ATC = C), genito-urinary drugs and sex hormones (ATC = G), nervous system drugs (ATC = N), and sensory organs medications (ATC = S) were significantly increased only in the first year of follow-up. Five years after diagnosis, CD subjects still had a higher risk of being prescribed most drug categories compared to their references (Additional file 2: Table S2).

**Table 4:** Risks of first drug prescription according to first level of Anatomical Therapeutic Chemical (ATC) Classification after date of diagnosis in CD subjects compared to matched references, stratified by first outcome occurrence (30–365 days after the index date/>365 days after the index date)^a^

First outcome occurrence	30–365 days					>1 year after index date^b^				
ATC codes^b^	REFERENCES		CD		HR (95 % CI)	REFERENCES		CD		HR (95 % CI)
	*n*	py	*n*	py		*n*	py	*n*	py	
A	131	5320	140	1129	**4.70** (3.66–6.04)	406	32774	158	6926	**1.78** (1.45–2.17)
B	48	5543	91	1534	**6.35** (2.93–13.75)	210	34991	102	7669	**2.36** (1.83–3.04)
C	18	5380	14	1222	**3.53** (1.71–7.29)	84	35296	29	8289	1.42 (0.91–2.21)
D	43	5367	17	1220	1.45 (0.79–2.64)	137	34694	38	8162	1.14 (0.77–1.68)
G	22	5375	14	1221	**2.38** (1.16–4.88)	104	35374	24	8408	0.97 (0.61–1.54)
H	209	5286	133	1157	**3.07** (2.45–3.85)	569	32328	183	6925	**1.65** (1.38–1.97)
J	1986	4300	524	941	**1.27** (1.15–1.41)	1674	13312	361	2739	**1.28** (1.10–1.49)
L	4	5386	12	1221	**12.46** (3.94–39.42)	15	35964	13	8476	**3.33** (1.52–7.29)
M	15	5383	14	1221	**3.64** (1.66–7.97)	155	35291	59	8214	**1.75** (1.27–2.41)
N	31	5368	16	1217	**2.22** (1.20–4.12)	125	35335	40	8316	1.35 (0.93–1.97)
P	44	5368	30	1211	**3.05** (1.88–4.94)	172	34891	60	8060	**1.73** (1.27–2.35)
R	808	4969	248	1097	**1.42** (1.23–1.65)	1251	22989	312	4907	**1.26** (1.09–1.45)
S	43	5368	25	1217	**2.29** (1.37–3.83)	104	34572	26	8035	1.03 (0.65–1.64)

Py: person–years; CD: celiac disease; HR: Hazard Ratio; CI: confidence interval

Figures in bold are statistically significant results (*p*-value <0.05)

^a^Matched by year of birth, gender and maternal education; analysis restricted to subjects with index date ≥1995 (because drug prescription data are available from that year); ^b^excluding subjects with less than one year of follow-up for each outcome

Among CD cases, 160 (12.4 %) were born to graduated mothers, 648 (50.1 %) to mothers with secondary school education and 471 (36.4 %) to less educated mothers (up to middle school) (15 cases had missing information on maternal education); by study design, references showed similar distributions, because each CD case was matched to references with the same maternal education.

In the analysis stratified by maternal education level, we found no clear modifying effect of maternal education (Additional file 3: Table S3, Additional file 4: Table S4). The risks of being hospitalized for any cause among CD subjects compared to their references born to mothers with similar maternal education were: HR 2.64 (95 % CI 1.79-3.89) for university level, HR 2.33 (95 % CI 1.97-2.76) for secondary school education and HR 2.30 (95 % CI 1.92-2.75) for primary/middle school education. Neither were there any differences in HRs for specific groups of causes according to maternal education although the low number of subjects with graduated mothers did not allow precise or computable estimates in that group (Additional file 3: Table S3). Similar risks were also seen for the majority of drug prescription categories in the groups of subjects stratified by maternal education (Additional file 4: Table S4).

## Discussion

In this large population-based birth cohort, we followed-up 1294 children and adolescents with CD and 5,681 peers without CD, matched by socio-demographic characteristics, for hospitalization and drug prescription after CD diagnosis. Both hospitalization and drug consumption were increased in CD cases in most disease categories and a wide range of drug classes.

A small number of previous studies have reported a greater use of medical services and drugs in CD patients compared to controls after diagnosis, [9–11] but only one included children and adolescents and provided a long follow-up [11]. To our knowledge, this is the first study on the long-term healthcare impact of paediatric CD conducted in Southern Europe. Healthcare use is influenced by culture and type of healthcare system, therefore it is worthwhile to conduct similar studies in different settings. Overall the results of the present study are consistent with current knowledge that childhood CD is associated with multi-organic complications and comorbidities and also with non-specific health complaints [2, 3].

For most disease and drug categories (in particular endocrine/metabolic/immune and digestive disorders and antineoplastic/immunomodulating agents), the risk of hospitalization or prescription was highest during the first year of follow-up, indicating that CD-related health problems are likelier to occur (or become recorded) shortly after diagnosis, when clinical surveillance is greatest and dietary treatment may have not yet displayed its effects. However, our findings suggest that CD-related health problems can develop also later, since for most conditions the risk of a first event after diagnosis was increased in CD subjects compared to reference individuals also after the first year of follow-up. To assess whether the increased healthcare use in CD persisted long-term, we also examined hospital admissions and drug prescriptions occurring five or more years after diagnosis; subjects who had hospital admissions or drug prescription within five years were included in this analysis. We found that even beyond five years of follow-up, the risk of hospitalization and drug prescription was greater in CD cases compared to reference individuals. Such results are consistent with a previous study conducted in the United Kingdom showing that healthcare costs in patients with CD have a peak around time of diagnosis and decline thereafter, but remain higher than in controls for up to 10 years [11]. Unlike the abovementioned study, we did not focused on costs but rather on the risk of events.

Different factors may explain why diagnosed CD children and adolescents used more healthcare resources than their references even five years after diagnosis. First of all, as in any chronic disorder, the frequent contact with the healthcare system can increase the chance of being diagnosed and subsequently treated for other conditions. In our opinion, however, surveillance bias alone cannot explain the differences we observed. Secondly, CD has been associated with non-specific health complaints such as functional gastrointestinal disorders and depressive and anxiety symptoms that can persist over time despite appropriate treatment [5, 6]. Consistently, when we examined main diagnoses and drug categories in detail, we found that anxiety and somatoform disorders (ICD9-CM 300), personality disorders (ICD9-CM 301), eating, sleep and psychogenic pain disorders (ICD9-CM 307), ill-defined symptoms concerning nutrition and abdomen (ICD9-CM 783 and 789), prescriptions of antacids (ATC A02), psycholeptics (ATC N05), and psychoanaleptics (ATC N06) were all more common among individuals with CD. Young CD patients may also be exposed to a persistent risk of developing complications of nutritional deficiencies due to incomplete remission of gut mucosal abnormalities or to dietary restrictions [14]. This is suggested by our finding that CD subjects had a higher proportion of iron-deficiency anemia (ICD9-CM 280), thrombocytopenia (ICD9-CM 287), leukocytopenia (ICD9-CM 288), and prescription of anti-anemic preparations (ATC B03). Infectious diseases, particularly those provoked by encapsulated bacteria, may also complicate the clinical course of CD in the long term, [15] as a result of hyposplenism [16] and perhaps of altered intestinal permeability and nutritional deficiencies. We also observed a greater frequency of hospital admissions for otitis media (ICD9-CM 381), pneumonia (ICD9-CM 481, 482, 485, 486), and urinary infections (ICD9-CM 590, 599) in CD, as well as a higher consumption of systemic anti-infectives (ATC J). Finally, a greater occurrence of several diseases, mainly but not exclusively of autoimmune origin, has been described in patients with CD. Although our study was not designed to estimate the risk of specific associated conditions, we think that some of the reported risks could be partially explained by underlying comorbidities. For instance, the greater proportion of admissions for diabetes (ICD9-CM 250) and prescription of antidiabetic agents (ATC A10) and thyroid drugs (ATC H03) could reflect the acknowledged associations between CD and type I diabetes mellitus and autoimmune thyroid disorders [17]. Similarly, the well-known comorbidity between CD and chromosomal anomalies, such as Down [18] and Turner syndrome, [3] may in part explain a higher prevalence of congenital malformations in CD subjects [19]. Several neurologic and psychiatric conditions, such as epilepsy (ICD9-CM 345) [20] and autism (ICD9-CM 299), [3, 21] have been linked to CD although with inconclusive results, and may explain our finding of a higher risk of hospital admission for neurologic and mental disorders and of prescription of antiepileptic drugs (ATC N03) in CD cases. Concerning digestive disorders, most of the increased risk in CD subjects is likely due to CD itself, especially in the first year of follow-up, since intestinal malabsorption (ICD9-CM 579) was not excluded from the analysis of hospitalization outcomes. Our CD cases also had a high frequency of hospitalizations for Crohn’s disease (ICD9-CM 555) and ulcerative colitis (ICD9-CM 556), both of which have been potentially linked to CD although earlier research has shown conflicting results [22–25].

Although CD is a chronic illness, many of its symptoms and effects can be reversed or controlled with a gluten-free diet [2]. Therefore, one might suppose that with a well-conducted gluten-free diet and an appropriate follow-up, the long-term outcome of CD paediatric patients would be similar to the general population ones. Our data showed that this is not the case, at least not in FVG Region. This issue deserves further investigation to confirm our findings and to assess whether the unfavorable long-term outcome of paediatric CD can be attributed only to complications and comorbidities that are not prevented by the gluten-free diet or also to an inadequate management of CD.

Socioeconomic factors in early childhood have a well-known impact on several health outcomes in adulthood, although evidence regarding early exposure impact on childhood and adolescence health is more limited [26]. Moreover, socio-economic level has been associated with the risk of developing CD in some studies, but results are conflicting [13, 27–32]. For this reason we decided to control for the possible confounding effect of socio-economic status by design matching reference individuals by maternal education. To the best of our knowledge this has never been done before. We also hypothesised that a poorer educational and socio-economic condition may adversely affect health of CD children and adolescents compared to reference individuals within the same socioeconomic stratum, possibly because of a poorer adherence to gluten-free diet. However, the analyses stratified by maternal education showed similar HR of hospital admission and drug prescription across groups. These results, although limited by the low number of events particularly for the hospitalization outcomes and among the most educated mothers, do not suggest a modifying effect of maternal education on the association between CD and healthcare use, but as any novel tested hypothesis they need to be further replicated in other setting/studies.

Strengths of our study besides the matched design include the large population size, the longitudinal design with long follow-up period and the availability of objective data, with no risk of recall bias. The main limitations are due to the use itself of administrative data with the inability to directly verify the presence of CD, and obtain data on compliance with a gluten-free diet. As regards the identification of CD cases, however, we are confident that our data are accurate since 71 % of cases had a biopsy report of villous atrophy, which has been found to be highly specific (positive predictive value 95 %) for CD in a validation study [33]. Of the remaining cases, 20 % had a copayment exemption, which in Italy is based on a physician’s diagnosis. Treatment with a gluten-free diet may result in decreased consumption of health care services and drugs, [10] although some studies suggested that, even after years of compliance with a gluten-free diet, CD patients have significantly impaired quality of life [11]. Moreover, we were unable to retrieve data on outpatient visits and diagnostic procedures, which would have drawn a more comprehensive picture of healthcare utilization. A further limitation is that we did not exclude from the analyses those subjects already affected by chronic disorders other than CD before the index date. Therefore, the higher healthcare use we observed in CD individuals may in part depend on comorbidities that were already present before the onset of CD. Our study should be therefore regarded as exploratory rather than confirmatory because we did not examine temporal relationships between CD onset and outcomes. The aim of this study was to describe healthcare use pattern after CD diagnosis without inferring causal relationships between CD and associated conditions.

## Conclusions

Using a population-based birth cohort matched study design, we showed that young people with diagnosed CD were at increased risk of later hospitalization and medication use compared to unaffected peers from the general population independently from socio-economic status at birth. Greater healthcare consumption may be explained both by non-specific health complaints and by complications and associated conditions of CD. Whatever the mechanism, our findings show that the burden associated with paediatric CD is heavy and persists in the long term, despite the existence of an effective treatment. Further longitudinal studies should be devoted to identify factors associated to a bad prognosis of CD in the paediatric population, in order to improve the management of this disease.

## Additional files

Additional file 1: Table 1. Risks of first hospital admission according to ICD9-CM classification five or more years from date of diagnosis in CD subjects compared to matched references*. (PDF 249 kb) Additional file 2: Table 2. Risks of first drug prescription according to first level of Anatomical Therapeutic Chemical (ATC) Classification five or more years from date of diagnosis in CD subjects compared to matched references*. (PDF 248 kb) Additional file 3: Table 3. Risks of first hospital admission according to ICD9-CM classification after date of diagnosis in CD subjects compared to matched references, stratified by maternal education*. (PDF 262 kb) Additional file 4: Table 4. Risks of first drug prescription according to first level of Anatomical Therapeutic Chemical (ATC) Classification after date of diagnosis in CD subjects compared to matched references, stratified by maternal education*. (PDF 257 kb)

## References

[1] Mustalahti K, Catassi C, Reunanen A, Fabiani E, Heier M, McMillan S (2010). The prevalence of celiac disease in Europe: results of a centralized, international mass screening project. Ann Med.

[2] Green PH, Jabri B (2003). Coeliac disease. Lancet.

[3] Newton KP, Singer SA (2012). Celiac disease in children and adolescents: special considerations. Semin Immunopathol.

[4] Tio M, Cox MR, Eslick GD (2012). Meta-analysis: coeliac disease and the risk of all-cause mortality, any malignancy and lymphoid malignancy. Aliment Pharmacol Ther.

[5] Turco R, Boccia G, Miele E, Giannetti E, Buonavolontà R, Quitadamo P (2011). The association of coeliac disease in childhood with functional gastrointestinal disorders: a prospective study in patients fulfilling Rome III criteria. Aliment Pharmacol Ther.

[6] Mazzone L, Reale L, Spina M, Guarnera M, Lionetti E, Martorana S (2011). Compliant gluten-free children with celiac disease: an evaluation of psychological distress. BMC Pediatr.

[7] Altobelli E, Paduano R, Gentile T, Caloisi C, Marziliano C, Necozione S (2013). Health-related quality of life in children and adolescents with celiac disease: survey of a population from central Italy. Health Qual Life Outcomes.

[8] Long KH, Rubio-Tapia A, Wagie AE, Melton LJ, Lahr BD, Van Dyke CT (2010). The economics of coeliac disease: a population-based study. Aliment Pharmacol Ther.

[9] Roos S, Wilhelmsson S, Hallert C (2011). Swedish women with coeliac disease in remission use more health care services than other women: a controlled study. Scand J Gastroenterol.

[10] Ukkola A, Kurppa K, Collin P, Huhtala H, Forma L, Kekkonen L (2012). Use of health care services and pharmaceutical agents in coeliac disease: a prospective nationwide study. BMC Gastroenterol.

[11] Violato M, Gray A, Papanicolas I, Ouellet M (2012). Resource use and costs associated with coeliac disease before and after diagnosis in 3,646 cases: results of a UK primary care database analysis. PLoS One.

[12] Marmot M, Allen J, Bell R, Bloomer E, Goldblatt P (2012). WHO European review of social determinants of health and the health divide. Lancet.

[13] Canova C, Zabeo V, Pitter G, Romor P, Baldovin T, Zanotti R (2014). Association of maternal education, early infections, and antibiotic use with celiac disease: a population-based birth cohort study in northeastern Italy. Am J Epidemiol.

[14] Penagini F, Dilillo D, Meneghin F, Mameli C, Fabiano V, Zuccotti GV (2013). Gluten-free diet in children: an approach to a nutritionally adequate and balanced diet. Nutrients..

[15] Ludvigsson JF, Olen O, Bell M, Ekbom A, Montgomery SM (2008). Coeliac disease and risk of sepsis. Gut..

[16] Di Sabatino A, Rosado MM, Cazzola P, Riboni R, Biagi F, Carsetti R (2006). Splenic hypofunction and the spectrum of autoimmune and malignant complications in celiac disease. Clin Gastroenterol Hepatol..

[17] Elfstrom P, Sundstrom J, Ludvigsson JF (2014). Systematic review with meta-analysis: associations between coeliac disease and type 1 diabetes. Aliment Pharmacol Ther.

[18] Marild K, Stephansson O, Grahnquist L, Cnattingius S, Söderman G, Ludvigsson JF (2013). Down syndrome is associated with elevated risk of celiac disease: a nationwide case-control study. J Pediatr..

[19] Wingren CJ, Agardh D, Merlo J (2012). Congenital anomalies and childhood celiac disease in Sweden. J Pediatr Gastroenterol Nutr.

[20] Ludvigsson JF, Zingone F, Tomson T, Ekbom A, Ciacci C (2012). Increased risk of epilepsy in biopsy-verified celiac disease: a population-based cohort study. Neurology..

[21] Ludvigsson JF, Reichenberg A, Hultman CM, Murray JA (2013). A nationwide study of the association between celiac disease and the risk of autistic spectrum disorders. JAMA Psychiatry.

[22] Yang A, Chen Y, Scherl E, Neugut AI, Bhagat G, Green PH (2005). Inflammatory bowel disease in patients with celiac disease. Inflamm Bowel Dis.

[23] Casella G, D'Inca R, Oliva L, Daperno M, Saladino V, Zoli G (2010). Prevalence of celiac disease in inflammatory bowel diseases: An IG-IBD multicentre study. Dig Liver Dis.

[24] Virta LJ, Kolho KL (2013). The risk of contracting pediatric inflammatory bowel disease in children with celiac disease, epilepsy, juvenile arthritis and type 1 diabetes--a nationwide study. J Crohns Colitis.

[25] El-Matary W, Senthilselvan A, Fedorak RN, Dieleman L, Spady D (2013). Celiac disease in children with inflammatory bowel disease: a prospective cohort study. Am J Gastroenterol.

[26] Spencer N, Thanh TM, Louise S (2013). Low income/socio-economic status in early childhood and physical health in later childhood/adolescence: a systematic review. Matern Child Health J.

[27] Kondrashova A, Mustalahti K, Kaukinen K, Viskari H, Volodicheva V, Haapala AM (2008). Lower economic status and inferior hygienic environment may protect against celiac disease. Ann Med..

[28] Roberts SE, Williams JG, Meddings D, Davidson R, Goldacre MJ (2009). Perinatal risk factors and coeliac disease in children and young adults: a record linkage study. Aliment Pharmacol Ther..

[29] Olen O, Bihagen E, Rasmussen F, Ludvigsson JF (2012). Socioeconomic position and education in patients with coeliac disease. Dig Liver Dis..

[30] Wingren CJ, Bjorck S, Lynch KF, Ohlsson H, Agardh D, Merlo J (2012). Coeliac disease in children: a social epidemiological study in Sweden. Acta Paediatr..

[31] Whyte LA, Kotecha S, Watkins WJ, Jenkins HR (2014). Coeliac disease is more common in children with high socio-economic status. Acta Paediatr..

[32] Zingone F, West J, Crooks CJ, Fleming KM, Card TR, Ciacci C (2015). Socioeconomic variation in the incidence of childhood coeliac disease in the UK. Arch Dis Child..

[33] Ludvigsson JF, Brandt L, Montgomery SM, Granath F, Ekbom A (2009). Validation study of villous atrophy and small intestinal inflammation in Swedish biopsy registers. BMC Gastroenterol..

