# Supplementary material for: Risks of hospitalization and drug consumption in children and young adults with diagnosed celiac disease and the role of maternal education: a population-based matched birth cohort study
Source: BMC Gastroenterol. 2016 Jan 5;16:1. doi: 10.1186/s12876-015-0415-y (PMC4722621; doi:10.1186/s12876-015-0415-y)
Supplement: Additional file 1: Table 1. — Risks of first hospital admission according to ICD9-CM classification five or more years from date of diagnosis in CD subjects compared to matched references*. (PDF 249 kb) [file 12876_2015_415_MOESM1_ESM.pdf]

Additional Table 1. Risks of first hospital admission according to ICD9-CM classification five or more years from date of diagnosis in CD subjects compared to matched references*

**ICD codes**	**REFERENCES (n=3900)**		**CD (n=777)**		**HR (95% CI)**
	**n**	**py**	**n**	**py**	
001-V89	520	39074	221	8774	**2.03** (1.71-2.41)
001-139	24	41370	15	9771	**2.45** (1.24-4.85)
140-239	32	41370	11	9783	1.44 (0.70-2.96)
240-279	42	41288	31	9671	**3.13** (1.94-5.06)
280-289	10	41441	5	9821	1.38 (0.38-5.06)
290-319	31	41393	15	9760	**1.99** (1.04-3.81)
320-389	36	41299	24	9706	**2.76** (1.62-4.72)
390-459	22	41431	11	9802	**2.39** (1.14-5.00)
460-519	75	40998	28	9725	1.37 (0.86-2.18)
520-579	102	41098	66	9497	**2.90** (2.09-4.02)
580-629	35	41332	15	9775	1.75 (0.92-3.31)
630-679	28	41435	10	9817	1.70 (0.82-3.52)
680-709	28	41380	9	9789	1.44 (0.67-3.07)
710-739	58	41298	17	9776	0.95 (0.51-1.80)
740-779	30	41352	20	9760	**2.93** (1.64-5.21)
780-799	35	41311	21	9772	**2.62** (1.50-4.61)
800-999	75	41491	26	9840	1.43 (0.90-2.27)
V01-V89	14	41433	14	9772	**4.87** (2.32-10.23)

Py: person-years; CD: celiac disease; HR: Hazard Ratio; CI: confidence interval

Figures in bold are statistically significant results (p-value <0.05)

*matched by year of birth, gender and maternal education
