# Supplementary material for: Risks of hospitalization and drug consumption in children and young adults with diagnosed celiac disease and the role of maternal education: a population-based matched birth cohort study
Source: BMC Gastroenterol. 2016 Jan 5;16:1. doi: 10.1186/s12876-015-0415-y (PMC4722621; doi:10.1186/s12876-015-0415-y)
Supplement: Additional file 2: Table 2. — Risks of first drug prescription according to first level of Anatomical Therapeutic Chemical (ATC) Classification five or more years from date of diagnosis in CD subjects compared to matched references*. (PDF 248 kb) [file 12876_2015_415_MOESM2_ESM.pdf]

Additional Table 2. Risks of first drug prescription according to first level of Anatomical Therapeutic Chemical (ATC) Classification five or more years from date of diagnosis in CD subjects compared to matched references*

ATC codes	REFERENCES (n=3505)		CD (n=698)		HR (95% CI)
	n	py	n	py	
A	241	40582	123	9377	**2.38** (1.88-3.00)
B	126	41081	72	9572	**2.85** (2.10-3.87)
C	35	41305	15	9755	**1.91** (1.03-3.55)
D	59	41216	16	9751	0.91 (0.49-1.71)
G	61	41280	15	9804	1.09 (0.61-1.93)
H	318	40293	126	9308	**1.83** (1.47-2.27)
J	1728	33646	435	7805	**1.16** (1.03-1.31)
L	10	41435	7	9808	2.55 (0.92-7.09)
M	94	41181	44	9690	**2.07** (1.41-3.04)
N	91	41122	31	9713	1.38 (0.89-2.13)
P	67	41204	23	9741	1.53 (0.93-2.51)
R	773	37873	228	8797	**1.34** (1.15-1.58)
S	18	41340	6	9788	1.11 (0.40-3.06)

Py: person-years; CD: celiac disease; HR: Hazard Ratio; CI: confidence interval

Figures in bold are statistically significant results (p-value <0.05)

*matched by year of birth, gender and maternal education; analysis restricted to subjects with index date ≥1995 (because drug prescription data are available from that year)
