# Supplementary material for: Risks of hospitalization and drug consumption in children and young adults with diagnosed celiac disease and the role of maternal education: a population-based matched birth cohort study
Source: BMC Gastroenterol. 2016 Jan 5;16:1. doi: 10.1186/s12876-015-0415-y (PMC4722621; doi:10.1186/s12876-015-0415-y)
Supplement: Additional file 3: Table 3. — Risks of first hospital admission according to ICD9-CM classification after date of diagnosis in CD subjects compared to matched references, stratified by maternal education*. (PDF 262 kb) [file 12876_2015_415_MOESM3_ESM.pdf]

Additional Table 3. Risks of first hospital admission according to ICD9-CM classification after date of diagnosis in CD subjects compared to matched references, stratified by maternal education*

	UNIVERSITY LEVEL					SECONDARY SCHOOL					PRIMARY/MIDDLE SCHOOL				
ICD codes	REFERENCES (n=631)		CD (n=160)		HR (95% CI)	REFERENCES (n=2935)		CD (n=648)		HR (95% CI)	REFERENCES (n=2050)		CD (n=471)		HR (95% CI)
	n	py	n	py		n	py	n	py		n	py	n	py	
001-V89	88	3289	50	702	**2.64** (1.79-3.89)	601	16047	233	2741	**2.33** (1.97-2.76)	540	13418	210	2413	**2.30** (1.92-2.75)
001-139	3	3622	2	1000	5.00 (0.70-35.50)	42	19491	20	4309	**2.24** (1.29-3.91)	28	17385	22	3998	**3.41** (1.91-6.07)
140-239	2	3643	2	1029	4.40 (0.61-31.65)	20	19631	12	4460	2.13 (0.97-4.70)	33	17548	13	4107	**1.64** (0.84-3.21)
240-279	4	3636	5	1022	6.25 (1.68-23.27)	42	19467	31	4312	**2.99** (1.84-4.87)	49	17434	32	3906	**3.08** (1.94-4.87)
280-289	1	3628	2	1002	6.53 (0.53-80.93)	12	19660	12	4433	**4.12** (1.81-9.40)	10	17644	10	4097	**4.18** (1.69-10.35)
290-319	4	3628	6	996	**5.55** (1.47-20.92)	33	19561	15	4444	**1.89** (1.00-3.60)	21	17622	14	4088	**3.13** (1.59-6.17)
320-389	10	3604	9	974	**2.85** (1.08-7.52)	54	19327	30	4293	**2.45** (1.54-3.89)	50	17348	17	4058	1.63 (0.94-2.85)
390-459	1	3644	2	1014	10.00 (0.91-110.3)	17	19666	8	4501	2.17 (0.93-5.09)	11	17692	6	4162	**3.13** (1.11-8.85)
460-519	20	3550	6	1001	0.98 (0.36-2.67)	157	18385	63	3963	**1.95** (1.44-2.64)	163	16053	52	3709	**1.42** (1.02-1.98)
520-579	15	3600	26	808	**8.32** (4.12-16.80)	110	19164	104	3694	**5.23** (3.92-6.97)	91	17079	101	3259	**5.73** (4.19-7.84)
580-629	6	3632	3	1006	0.96 (0.11-8.24)	46	19446	25	4326	**2.62** (1.59-4.33)	41	17389	13	4115	1.27 (0.65-2.49)
630-679	1	3642	3	1028	**13.46** (1.40-129.72)	22	19675	3	4519	0.68 (0.20-2.27)	23	17675	9	4170	1.66 (0.76-3.64)
680-709	4	3636	0	1032	- (-)	23	19625	8	4479	1.36 (0.57-3.20)	27	17561	8	4121	1.307 (0.58-2.92)
710-739	4	3631	3	1019	3.08 (0.68-14.00)	51	19530	10	4474	0.66 (0.30-1.45)	48	17497	14	4134	0.935 (0.47-1.87)
740-779	5	3615	4	1012	2.58 (0.61-10.99)	47	19434	25	4319	**2.39** (1.44-3.95)	38	17451	14	4065	1.74 (0.94-3.24)
780-799	7	3624	3	1014	1.31 (0.27-6.32)	39	19480	30	4306	**3.48** (2.12-5.71)	36	17395	22	4004	**2.78** (1.60-4.81)
800-999	12	3646	7	1032	1.78 (0.65-4.88)	72	19714	23	4530	1.34 (0.82-2.19)	82	17729	26	4189	1.26 (0.79-2.01)
V01-V89	3	3627	5	996	**5.00** (1.01-24.78)	24	19602	18	4390	**3.17** (1.68-5.95)	30	17465	17	4001	**2.49** (1.35-4.61)

Py: person-years; CD: celiac disease; HR: Hazard Ratio; CI: confidence interval

Figures in bold are statistically significant results (p-value <0.05)

*matched by year of birth, gender and maternal education
