# Supplementary material for: Risks of hospitalization and drug consumption in children and young adults with diagnosed celiac disease and the role of maternal education: a population-based matched birth cohort study
Source: BMC Gastroenterol. 2016 Jan 5;16:1. doi: 10.1186/s12876-015-0415-y (PMC4722621; doi:10.1186/s12876-015-0415-y)
Supplement: Additional file 4: Table 4. — Risks of first drug prescription according to first level of Anatomical Therapeutic Chemical (ATC) Classification after date of diagnosis in CD subjects compared to matched references, stratified by maternal education*. (PDF 257 kb) [file 12876_2015_415_MOESM4_ESM.pdf]

Additional Table 4. Risks of first drug prescription according to first level of Anatomical Therapeutic Chemical (ATC) Classification after date of diagnosis in CD subjects compared to matched references, stratified by maternal education*

	**UNIVERSITY LEVEL**					**SECONDARY SCHOOL**					**PRIMARY/MIDDLE SCHOOL**				
**ATC codes***	**REFERENCES (n=607)**		**CD (n=153)**		**HR (95% CI)**	**REFERENCES (n=2816)**		**CD (n=617)**		**HR (95% CI)**	**REFERENCES (n=1878)**		**CD (n=430)**		**HR (95% CI)**
	**n**	**py**	**n**	**py**		**n**	**py**	**n**	**py**		**n**	**py**	**n**	**py**	
A	34	3484	28	892	**3.42** (1.95-5.98)	265	18097	143	3687	**2.52** (2.05-3.16)	234	16123	124	3403	**2.39** (1.88-3.04)
B	22	3561	24	934	**4.62** (2.47-8.65)	134	19150	84	4107	**3.35** (2.51-4.46)	102	17242	83	3728	**3.80** (2.77-5.21)
C	4	3621	4	1000	**4.00** (97-16.42)	51	19312	18	4424	1.61 (0.93-2.8)	47	17341	21	3997	**1.74** (1.01-3.01)
D	12	3579	4	1011	1.26 (0.40-4.03)	84	19119	24	4346	1.32 (0.82-2.12)	82	16972	26	3937	1.08 (0.66-1.79)
G	8	3614	4	999	1.07 (0.22-5.05)	54	19457	16	4459	1.17 (0.66-2.1)	64	17276	18	4081	1.28 (0.75-2.21)
H	74	3329	28	880	**1.85** (1.17-2.94)	385	17907	151	3720	**1.97** (1.61-2.4)	307	16040	132	3424	**2.20** (1.77-2.74)
J	379	1812	108	384	**1.42** (1.11-1.82)	1842	8230	422	1759	**1.20** (1.06-1.35)	1383	7471	344	1509	**1.36** (1.18-1.56)
L	0	3646	3	1020	- (-)	6	19660	14	4442	**10.41** (3.95-27.47)	13	17641	8	4146	2.36 (0.93-5.96)
M	13	3592	10	966	2.06 (0.80-5.27)	83	19283	36	4352	**2.11** (1.40-3.18)	74	17395	27	4028	**1.71** (1.07-2.73)
N	10	3606	6	1007	2.03 (0.66-6.26)	64	19362	26	4404	**1.73** (1.08-2.79)	80	17336	23	4034	1.29 (0.80-2.08)
P	26	3539	8	972	1.37 (0.59-3.18)	105	19140	42	4303	**2.05** (1.41-2.98)	83	17189	40	3907	**2.22** (1.49-3.38)
R	186	2701	61	694	**1.40** (1.21-1.62)	1014	13487	268	2726	**1.23** (1.04-1.45)	833	11505	223	2535	**1.42** (1.02-1.99)
S	13	3559	4	998	1.08 (0.34-3.46)	64	19086	22	4285	1.44 (0.86-2.41)	70	16893	25	3879	1.48 (0.91-2.4)

Py: person-years; CD: celiac disease; HR: Hazard Ratio; CI: confidence interval

Figures in bold are statistically significant results (p-value <0.05)

*matched by year of birth, gender and maternal education; analysis restricted to subjects with index date ≥1995 (because drug prescription data are available from that year)
